# Ventilation and Air Quality in Student Dormitories in China: A Case Study during Summer in Nanjing

**DOI:** 10.3390/ijerph15071328

**Published:** 2018-06-25

**Authors:** Zhe Yang, Jialei Shen, Zhi Gao

**Affiliations:** School of Architecture and Urban Planning, Nanjing University, 22 Hankou Road, Nanjing 210093, China; zhyang@smail.nju.edu.cn (Z.Y.); jialeishen@smail.nju.edu.cn (J.S.)

**Keywords:** PM2.5, ozone, student dormitory, I/O ratio, ventilation, indoor air quality

## Abstract

The Air quality in student dormitories can have a major impact on the health of millions of students in China. This study aims to investigate the ventilation and air quality in student dormitories. Questionnaire survey was conducted in eight dormitory buildings and field measurements were conducted in one dormitory during the summer in Nanjing. The survey result reveals that most students thought the indoor and outdoor air quality was neutral and the correlation between indoor and outdoor perceived air quality is statistically significant. There are few indoor PM2.5 and ozone sources in dormitories and natural ventilation is the most common form of ventilation. However, there is no statistically significant correlation between window opening behaviors and the perceptions of indoor and outdoor air quality. The field measurement result shows the measured I/O ratios of PM2.5 and ozone over 37 days are in the range of 0.42–0.79 and 0.21–1.00, respectively. The I/O ratios for PM2.5 and ozone are 0.49 ± 0.05 and 0.26 ± 0.05 in the case of the window being closed, and the I/O ratios for PM2.5 and ozone are 0.65 ± 0.08 and 0.50 ± 0.15 in the case of the window being open. The outdoor and indoor ozone concentrations show pronounced diurnal periodic variations, while the PM2.5 concentrations do not. Finally, recommended open/close window strategies are discussed to reduce indoor pollutant levels. Understanding the indoor/outdoor PM2.5 and ozone concentrations in different window patterns can be a guidance to preventing high indoor PM2.5 and ozone exposure in student dormitories.

## 1. Introduction

Air pollution is a public health risk that many modern cities face, particularly the cities in developing countries like China. In recent years, events related with air pollution have been frequently observed in China. Fine particulate matter (PM2.5) and ozone are usually the main air pollutants. Numerous studies have suggested that PM2.5 and ozone may adversely affect people’s health and result in an increase in respiratory morbidity [1,2,3,4], impaired lung function [5,6,7,8,9,10], local or systemic inflammation [3,11,12,13], cardiovascular disease [6,7,10] and cancer [14,15,16,17,18,19,20,21,22,23,24,25]. Indoor air quality is significant for occupants’ health since most people spend approximately 90% of their time indoors [26,27,28,29]. Outdoor PM2.5 and ozone concentrations can be a major contributor to indoor concentrations [30,31]. Indoor ozone concentrations in buildings are generally below 50 ppb [32] while indoor PM2.5 concentrations vary considerably (from less than 10 μg/m^3^ up to more than 600 μg/m^3^) depending on both indoor and outdoor sources [33]. The indoor/outdoor (I/O) ratio, affected by the permeability of the building envelope, air change rate, decay rate of pollutants and indoor pollutant sources, indicates the relationship between indoor and outdoor PM2.5 and ozone concentrations [34]. Weschler [34] revealed that the I/O ratios of ozone for most buildings in the United States were in the range of 0.2–0.7. Chen and Zhao [31] presented a detailed review about the relationship between indoor and outdoor particles across different countries and indicated that I/O ratios in different studies vary in a large range (from 0.02 to 31) owing to differing measurement conditions. Zhao et al. [35] reported that the I/O ratios of PM2.5 during winter in Beijing were mainly distributed between 0.4 and 0.9. Shao et al. [36] revealed that I/O ratios of PM2.5 in residential buildings in different seasons in Nanjing ranged from 0.36 to 4.76.

**Figure 1:**
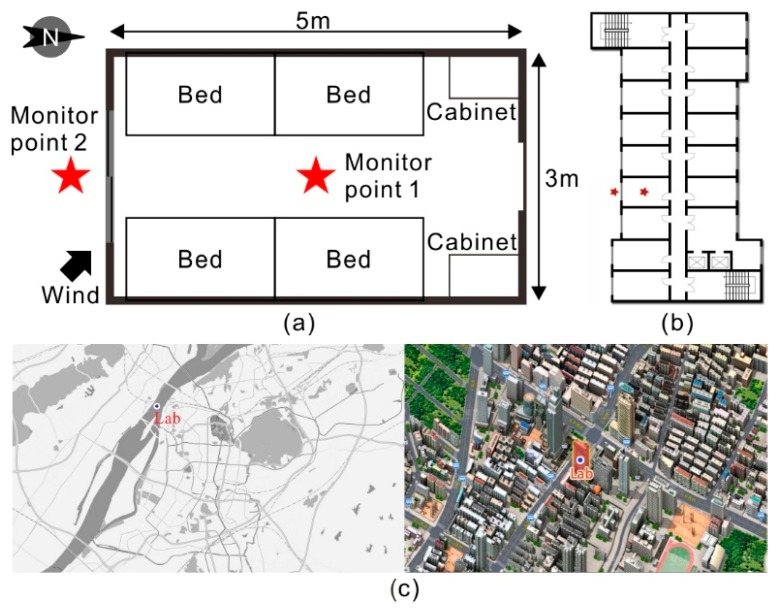
(**a**) A typical student dormitory in China; (**b**) Location floor of the student dormitory; (**c**) Location of the monitored field.

The student dormitory is a typical kind of residential building in the vast majority of colleges in China. In recent years, the number of students enrolled in colleges in China has notably increased, most of which have lived in campus dormitories. The statistical results show that there were approximately 15 million undergraduate students and 1.8 million graduate students residing in dormitories in 2014 [37]. Dormitories in China generally have highly homogeneous indoor layouts, building materials and furniture. Compared to residential houses, student dormitories are furnished more simply and have less fewer electrical appliances [38]. Each dormitory room usually houses four students and contains four loft beds situated over top of desks with high residential density as shown in Figure 1. Natural ventilation through the windows is generally the most common ventilation form in dormitories since few of the dormitories have a mechanical ventilation system or device (i.e., an exhaust fan). Air quality in dormitories is closely related to millions of students’ health because they have to live in dormitories for four or more years. Some previous studies focused on the air quality in student dormitories in China. Li et al. [39] conducted large-scale field measurements of natural ventilation in college student dormitories in Beijing. Pei et al. [40] monitored the long-term gas pollutants concentrations (i.e., formaldehyde, TVOC and ammonia) during a 3 month period in 14 unoccupied newly built student dormitories in a new campus in Tianjin. Li et al. [38] presented the students’ exposure to phthalates in dormitories and studied phthalates in dormitory dust from three northern Chinese cities. Chen et al. [41] analyzed the concentrations, sources and health risk assessments of PM2.5-bound PAHs in a dormitory in Beijing across four seasons. Liu et al. [42] investigated PM2.5, TVOCs and bioaerosols in 30 student dormitories in a university campus in Tianjin. While these studies have examined air quality, natural ventilation, gas pollutant concentrations (i.e., formaldehyde, TVOC and ammonia), and phthalates, investigations on indoor/outdoor PM2.5 and ozone concentrations in dormitories in China are still scarce.

This study focuses on ventilation and indoor air quality in a student dormitory in China. A questionnaire survey was conducted in the summer of 2013 to figure out student behavior patterns in the dormitories and the relationship of these behaviors to indoor PM2.5 and ozone sources/sinks and ventilation patterns. The concentrations of indoor and outdoor PM2.5/ozone in a student dormitory in Nanjing were monitored from July to August in the summer of 2015, and the window opening behaviors were recorded during the measurements. The questionnaire survey and field measurements were conducted in the summer because of the high ambient PM2.5 and ozone concentrations during this period [43,44]. The relationships between indoor and outdoor concentrations were analyzed using the I/O ratio, air change rate and ventilation patterns. The PM2.5 and ozone concentration in the dormitory could be inferred from the I/O ratio under a different window opening status by the different outdoor PM2.5 and ozone concentration. Some guidance was given to the window opening behavior of the dormitory to reduce indoor exposure to PM2.5 and ozone. Understanding the indoor/outdoor PM2.5 and ozone concentrations in different window patterns can help prevent high indoor PM2.5 and ozone exposure in student dormitory by advising when the students should open/close the window.

## 2. Methodology

### 2.1. Questionnaire Survey

A questionnaire survey associated with ventilation and air quality about their daily perceptions and behavior was conducted in 8 dormitory buildings on the campus of Nanjing University (i.e., No. 2, 5, 6, 8, 9, 12, 13 and 15 Dormitory Buildings) during the summer of 2013. Ten dormitories were selected randomly on each floor of each dormitory building to perform the survey. The questions in the questionnaire were mainly related to the occupant perceptions and behavior patterns in the dormitory, including but not limited to the questions listed in Table 1, of which questions 1 to 6 were single option questions while question 7 was a multiple option question.

### 2.2. Field Measurement

Field measurements were conducted on non-rainy days from 15 July to 31 August 2015 in one student dormitory on the campus of Nanjing University, of which 37 days of data were available. Window opening statuses were strictly controlled and the weather conditions were recorded. The room volume of the studied dormitory was approximately 45 m^3^ (3 m × 5 m × 3 m), without a balcony or bathroom inside the room. The studied dormitory contained four loft beds situated over top of desks and two students (volunteers) lived in the dormitory during the test period, whose daily activities were not disturbed by the experiments. There was no ozone generating devices such as a photocopier, printer and ozone generator or particulate source that comes from smoking or cooking in the dormitory. Therefore, it is believed that there was no any crucial indoor ozone and PM2.5 source in the dormitory, since the above-mentioned devices or activities were considered to be the major indoor sources of ozone and PM2.5 [45,46,47,48,49,50,51,52]. In addition, there was no air cleaner in the dormitory. An openable sliding window was mounted on the room wall, with an openable area of 0.84 m^2^. The operation patterns (open or closed) of the window were recorded manually by these two student dormitories every hour in the studied dormitory.

The concentrations of indoor and outdoor PM2.5 and ozone were simultaneously monitored every minute during the study period. Indoor and outdoor ozone concentrations were sampled by 2B Technologies Model 202 and POM monitors, respectively. Two TSI DUSTTRAK II-Model 8532 monitors were used to measure the indoor and outdoor PM2.5 concentrations. All of the monitor instruments were calibrated within one year by the manufacturers. The indoor sampling point was located at the center of the room with a vertical height of 1.5 m, i.e., breathing zone height, while the outdoor sampling point was located at the outside of the window. The tracer gas decay method was used to measure the air change rate (ACH) of the dormitory. Common tracer gases used for these measurements include nitrogen dioxide (NO_2_), sulphur hexafluoride (SF_6_) and carbon dioxide (CO_2_). In this study, SF_6_ was released inside the dormitory as the tracer gas. The initial concentration of the SF_6_ is 149 ppb, the air in dormitory was mixed well by fan. When the indoor SF_6_ concentration was stable (30 ppb), the concentrations during the decay period was monitored by a Innova 1412i Lumasense gas monitor under two different window opening statuses, i.e., window open and window closed. The sampling point was located at the center of the room with a vertical height of 1.5 m. The air change rate was calculated by the decay of SF_6_ concentration and the decay period. The air change rate of different window open statues includes inner penetration, but the air change rate mainly depends on the outdoor weather.

### 2.3. Data Analysis

It should be mentioned that there is a measure of subjectivity in the questionnaire about the air quality. The obtained questionnaire data were analyzed by Spearman correlation test to quantitatively represent the relationship between the occupant perception and different behaviors in the dormitory. The hour average of PM2.5 and ozone concentrations, which were recorded every minute during the monitoring period were calculated as the arithmetic mean of the time. The hourly I/O ratios of PM2.5 and ozone in different ventilation conditions were analyzed since I/O ratios can directly represent the relationship between indoor and outdoor pollutant concentrations. The relationship between indoor and outdoor pollutant concentrations in different ventilation conditions were discussed using linear regression analysis.

## 3. Results and Discussion

### 3.1. Questionnaire Survey

Totally 353 valid questionnaires were obtained, including 177 male dormitories and 176 female dormitories. All questions in the questionnaire were related to indoor air quality. Among them, question 1 and question 2 were related to the indoor and outdoor perceived air quality. No. 3 and No. 4 questions were about the potential pollutant source or sink in the dormitory. Questions 5, 6 and 7 were designed to figure out the ventilation patterns in the dormitory.

#### 3.1.1. Perceived Air Quality

**Figure 2:**
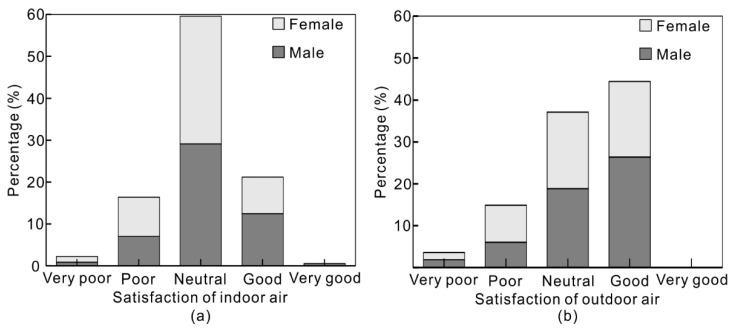
(**a**) Indoor perceived air quality; (**b**) Outdoor perceived air quality.

Figure 2 shows the indoor and outdoor perceived air quality. The questionnaire data depends on people’s subjective judgment and is unreliable to some extent. Most surveyed students, around 60%, considered the indoor air quality to be neutral. Another 22% of students thought that the indoor air quality was good or very good. Only 19% students thought the indoor air quality was poor or very poor. By contrast, more students thought the outdoor air quality was better relative to indoor air quality. 44% of the surveyed students regarded the outdoor air quality as good while no one thought it was very good, 22% higher compared to the data for indoor air quality. Only 37% of students thought the outdoor air quality was neutral, lower compared to the data for indoor air quality, and 18% of students thought it was poor or very poor. It indicates that the indoor and outdoor perceived air quality percentage of very poor and poor are very close (below 20%). In addition, the feeling of students tends towards neutral in the dormitory, while tending towards feeling good outdoors. Male surveyed students had higher evaluations of outdoor and indoor air quality than female students did. This indicates that men are more satisfied with their environment.

The relationship between indoor and outdoor perceived air quality was analyzed using a Spearman correlation test. The correlation between occupant perceptions to indoor and outdoor air quality is statistically significant (*p* value < 0.01). The correlation coefficient *r* is 0.529, which indicates that the indoor perceived air quality is moderately related to the outdoor perceived air quality.

#### 3.1.2. Indoor Sources/Sinks 

**Figure 3:**
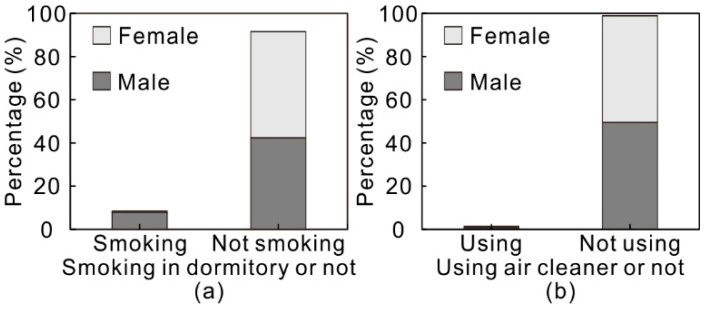
(**a**) Smoking in dormitory or not; (**b**) Using air cleaner in dormitory or not.

The sources of indoor PM2.5 consist of indoor generation and outdoor infiltration. Indoor PM2.5 sources mainly include smoking, cooking, fuel combustion for heating and human activities, of which smoking and cooking are usually more significant [47]. However, owing to the fact that kitchen and heating stoves are scarce in student dormitories in China, PM2.5 emitted from cooking and fuel combustion is generally negligible in student dormitories. Therefore, smoking may be the primary indoor PM2.5 source in student dormitories, which was surveyed in this study. According to the results shown in Figure 3a, smoking in the dormitory was found to occur in 8% of the surveyed dormitories. The smoking conditions between male and female dormitories vary significantly. Smoking was observed in only 1% female dormitories, while this proportion reached 15% for male dormitories. It can be seen that there is a significant gender difference in smoking as a PM2.5 source in the dormitories. Therefore, we can conclude that smoking may be an important potential indoor PM2.5 source in male dormitories. However, according to Spearman correlation test, the correlation between the smoking condition in the dormitories and the indoor perceived air quality is statistically insignificant (*p* value > 0.05), which is probably due to the limited smoking sample size (N = 29). Besides, occupant activities may be another significant PM2.5 source in student dormitories, particularly considering the high occupant density in dormitories.

Indoor ozone sources mainly include laser printers, photocopiers and ionization/ozonolysis air cleaners [53,54,55,56,57,58,59,60,61,62,63,64,65,66,67,68,69,70,71,72,73,74,75,76,77,78]. Particularly, ionization/ozonolysis air cleaners may be the principal indoor ozone source owing to the higher ozone emission rate [32]. However, all these ozone emission devices are rare in student dormitories. Devices like laser printers and photocopiers were not surveyed in this study while air cleaners were surveyed, although the working mechanisms of the air cleaners (e.g., filtration or ionization et al.) were not studied. According to the survey result in Figure 3b, within all the surveyed dormitories, there were only 3 dormitories that had air cleaners, including 2 male dormitories and 1 female dormitory. This indicates that using an air cleaner in a student dormitory is rare and thus indoor ozone sources can be negligible. In the absence of indoor ozone emission sources, ambient outdoor ozone entering into buildings is the primary source of indoor ozone.

Air cleaners can effectively remove indoor particles and thus is are prominent kind of indoor PM2.5 sink. The survey result in Figure 3b indicates that indoor PM2.5 sinks can be negligible for most student dormitories. In addition to air cleaners, indoor building materials like carpet, brick, activated carbon, even human skin can be significant indoor ozone sinks owing to their high ozone reactivity [79]. However, materials like carpet, brick and activated carbon are not commonly used in student dormitories. Considering the high occupant density in student dormitory, human skin is probably the main indoor ozone sink.

In conclusion, according to the questionnaire survey, there are few indoor PM2.5 and ozone sources in most student dormitories. The possible PM2.5 sources in some dormitories include smoking and occupant activities. Besides, the indoor PM2.5 and ozone sinks are considered to be negligible in dormitories. Indoor ozone removal by human skin may be the primary ozone sink in student dormitories. Therefore, the number of students in the dormitories and their behavior s are probably the determinant factor influencing the indoor PM2.5 and ozone emission and removal in the dormitories.

#### 3.1.3. Ventilation Patterns

**Figure 4:**
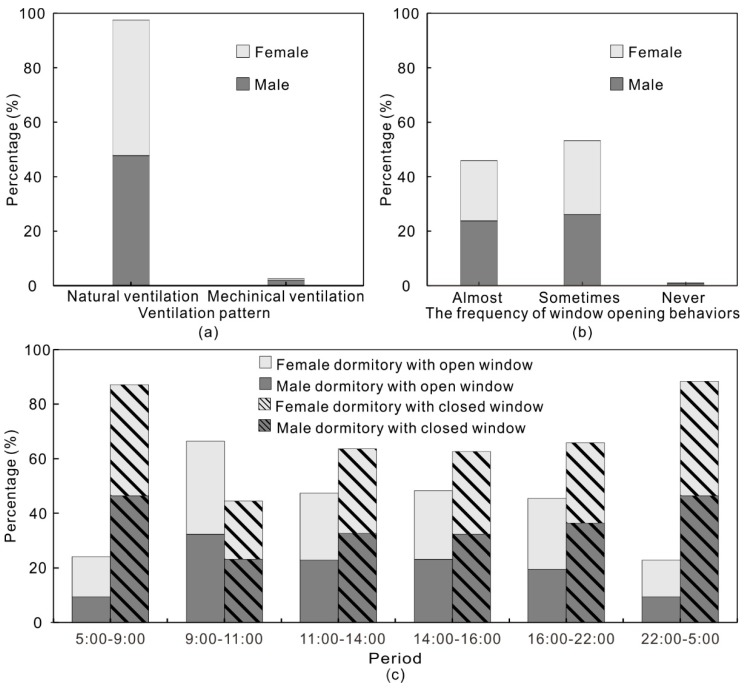
(**a**) Ventilation pattern in surveyed dormitories; (**b**) The frequency of window opening behaviors in dormitory; (**c**) The Period of window opening behaviors in dormitory.

Ambient PM2.5 and ozone entering into the dormitories is the primary source of indoor PM2.5 and ozone since the indoor sources are negligible in student dormitories. Therefore, the ventilation pattern of student dormitories is closely associated with indoor PM2.5 and ozone concentrations. The survey result in Figure 4a shows that natural ventilation is the most common ventilation pattern in the surveyed dormitories, approximately accounting for 97% of ventilation patterns. Therefore, window opening behaviors (open or closed) may be a direct factor affecting natural ventilation in the dormitories. The survey results of the frequency of window opening behaviors in dormitories are illustrated in Figure 4b. Most students open their windows sometimes (53%) or almost all day (46%), while only 1% of the surveyed students almost never open their windows. According to the Spearman correlation test, there is no statistically significant correlation between the frequency of window opening behaviors and indoor and outdoor perceived air quality during the survey period (*p* value > 0.05). Therefore, the frequency of window opening behaviors in dormitories in the summer is not statistically associated with the occupant perception of indoor and outdoor air quality. Other factors like temperature or relative humidity may be the possible factors affecting the frequency of window opening behaviors in student dormitories in the summer, which requires further investigation.

The period of window opening behaviors in dormitory in a day is shown in Figure 4c. The time of a day is divided into 6 periods. In essence, most students tend to close the windows in their dormitories during the sleeping period in the summer (from 22:00 to 9:00). Besides, more students are willing to open the windows in the dormitories during daytime and early evening (from 9:00 to 22:00). Particularly, the period between 9:00 to 11:00 is the period with the highest frequency of open windows in student dormitories during a day. Considering the fact that the students are always get up between 9:00 to 11:00, we can conclude that in summertime the students living in the dormitories tend to open the windows after waking up.

### 3.2. Field Measurement

#### 3.2.1. Concentrations in Time Sequence

**Figure 5:**
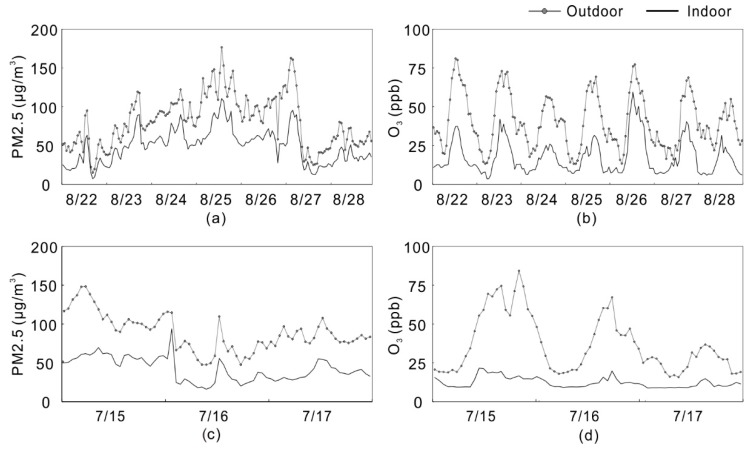
(**a**) Indoor and outdoor concentrations of PM2.5 during 7 days (22–28 August 2015) in the case of window open; (**b**) Indoor and outdoor concentrations of ozone during 7 days (22–28 August 2015) in the case of window open; (**c**) Indoor and outdoor concentrations of ozone during 3 days (15–17 July 2015) in the case of window closed; (**d**) Indoor and outdoor concentrations of ozone during 7 days (15–17 July 2015) in the case of window closed.

In total, 888 sets of available data were obtained, including 350 sets in window open and 538 sets in window closed. The concentrations of indoor and outdoor PM2.5 and ozone were recorded every minute. Indoor and outdoor concentrations of PM2.5 and ozone during 7 days (22–28 August 2015) in the case of window open are shown in Figure 5a,b, respectively. The indoor concentrations of PM2.5 and ozone are generally lower than the outdoor concentrations, and the indoor concentrations are closely related to the outdoor concentrations. Figure 5a shows indoor PM2.5 concentrations during this period are approximately between 10 and 120 µg/m^3^ and the outdoor PM2.5 concentrations are between 15 and 180 µg/m^3^. Figure 5a shows that the indoor PM2.5 concentrations vary with the outdoor concentrations almost simultaneously, although the indoor levels are always lower than the outdoor levels. Figure 5b shows that indoor ozone concentrations approximately range from 5 to 65 ppb while outdoor ozone concentrations range from 10 to 85 ppb. The outdoor ozone concentrations show prominently diurnal periodic variations, i.e., the rapid increasing of concentrations in the morning, reaching the peak at noon or afternoon, and then continuing to decline throughout the evening until reaching a low point at midnight, which have been presented by the literature [80,81,82,83]. The indoor ozone concentrations in the case of window open status also show diurnal periodic variations varied with the outdoor concentrations. However, the PM2.5 concentrations don’t show significant periodic variations during the study period. Figure 5 shows when outdoor concentration appears to be low, the indoor concentration would be close to and the trend of ozone is more significant than PM2.5.

Figure 5c,d illustrate indoor and outdoor concentrations of PM2.5 and ozone during 3 days (15–17 July 2015) in the case of having the window closed. In this case, the indoor concentrations are still closely related to the outdoor concentrations. However, the indoor concentrations of PM2.5 and ozone show more gentle variations than the case of having the window open, which leads to lower indoor concentrations in general. Therefore, the diurnal periodic variations of indoor ozone concentrations are not as prominent as the case of window open. Especially for ozone, the peak concentration of outdoor ozone is 3 times larger than the indoor concentration. It indicates that closed-window is an effective way to reduce the indoor ozone.

**Figure 6:**
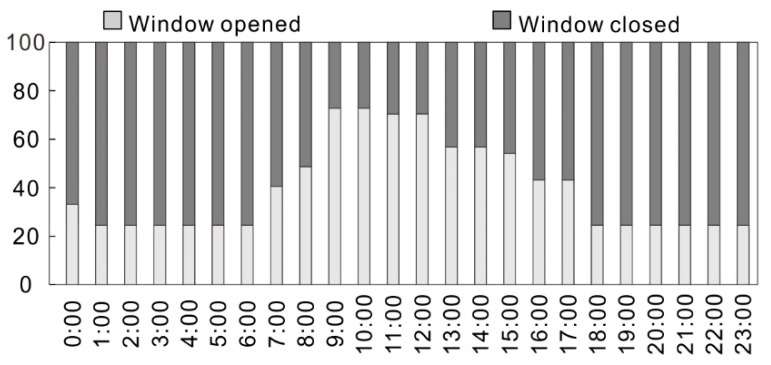
The frequency of opening window at different time.

Figure 6 shows the frequency of window opening behavior at different times during the 37 day test period. The period between 9:00 to 15:00 is the period with a higher frequency of windows being open for the studied dormitory, particularly during the period between 9:00 to 12:00. This recorded result is generally consistent with the questionnaire survey result in Section 3.1.3.

**Figure 7:**
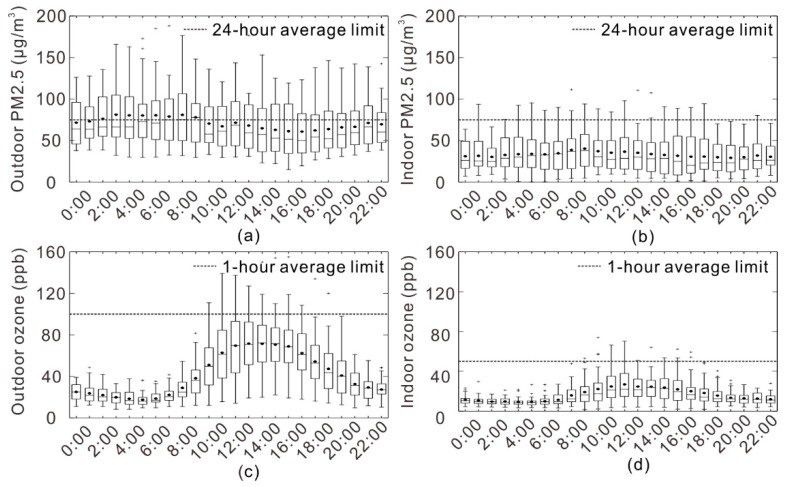
(**a**) Outdoor PM2.5 levels at different time periods during the 37 day experiment period; (**b**) Indoor PM2.5 levels at different time period during the 37 day experiment period; (**c**) Outdoor ozone levels at different time period during the 37 day experiment period; (**d**) Indoor ozone levels at different time period during the 37 day experiment period.

Figure 7 reveals the measured outdoor and indoor PM2.5 and ozone levels at different times during the 37 day test period and the dashed line indicates the limits value of outdoor and indoor PM2.5 and ozone concentration. As for PM2.5, there is no regulation limiting its concentration in China and the value was limited in this article as the ambient air quality standard is 75 μg/m^3^ for an average 24-h period [84]. The average 1-h limit for ozone outdoor and indoor is 100 ppb and 50 ppb, respectively [84,85]. The average outdoor PM2.5 levels during the 37 day experiment period is slightly less than the 24-h limit (75 μg/m^3^), but the average concentration is slightly larger than the limit value during 3:00 to 9:00. It indicated that the outdoor pollutant concentration was larger during this time than during the other time, so students would ideally remain in the dormitory during this time. For ozone limit regulation, the outdoor and indoor 1-h average limits are 100 ppb and 50 ppb, respectively. More than 75% of the outdoor and indoor ozone concentration during different period during the 37 days did not exceed the limit. The maximum experiment value of outdoor ozone was larger than the limit at noon and the maximum experiment indoor ozone concentration was larger than the limit during 11:00 to 12:00. Indoor concentration levels are generally lower than the outdoor levels and the situation that indoor concentration exceeds the limit is far less than outdoors, especially for PM2.5. The outdoor and indoor ozone concentrations show prominent diurnal periodic variations. The ozone concentrations during the daytime are significantly higher than the concentrations during the night. The outdoor and indoor ozone concentrations reach the highest concentration at noon, i.e., around 12:00 to 15:00. However, the outdoor and indoor PM2.5 levels do not show such significant diurnal variations. The outdoor PM2.5 concentrations during midnight and early morning, i.e., approximately 3:00 to 9:00, are slightly higher than the concentrations during afternoon, i.e., around 16:00 to 18:00. For indoor PM2.5, this kind of diurnal concentration variation is negligibly small.

The survey result reveals that most students often open their windows between 9:00 to 12:00. The outdoor ozone is at a high level around 40–80 ppb on 10:00 to 12:00 and the highest outdoor ozone concentration can reach 140 ppb. The daily concentration of PM2.5 is stable and its average is 75 μg/m^3^ between 9:00 and 12:00 and the highest value is close to 150 μg/m^3^. Therefore, the ozone concentration is the main problem to be considered. During 9:00 to 12:00, the mean values of indoor concentrations in window closed status are generally lower than the mean indoor concentrations in window open status, particularly for ozone. It indicates that student should stay indoors and minimize window opening in summer between 10:00 and 12:00. The outdoor and indoor ozone concentrations is at a low level at 7:00 to 8:00 and it is most advantageous time to open the window during this time for reducing indoor exposure to severe outdoor ozone pollution in the summer.

#### 3.2.2. Hour Average of PM2.5 and Ozone Concentrations in Different Window Opening Statuses

**Figure 8:**
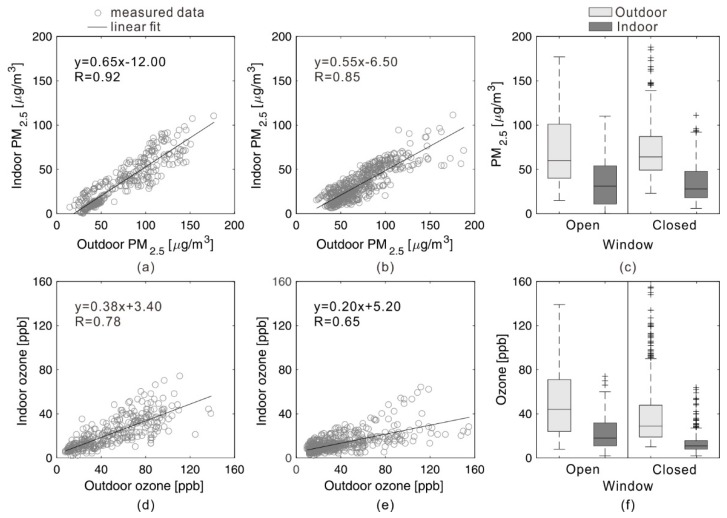
(**a**) Indoor and outdoor concentrations of PM2.5 in the cases of window open status; (**b**) Indoor and outdoor concentrations of PM2.5 in the cases of window closed status; (**c**) The PM2.5 I/O ratio in the cases of window open and closed statuses; (**d**) Indoor and outdoor concentrations of ozone in the cases of window open status; (**e**) Indoor and outdoor concentrations of ozone in the cases of window closed status; (**f**) The ozone I/O ratio in the cases of window open and closed statues.

The hour average of PM2.5 and ozone concentrations was calculated as the arithmetic mean value of the time sequence of PM2.5 and ozone concentrations every hour. Figure 8 reveals the indoor and outdoor concentrations of PM2.5 and ozone in the cases of different window opening statuses, i.e., window open or window closed. For both window opening statuses, indoor PM2.5 and ozone concentrations are lower than outdoor concentrations. In the window open status, the mean indoor ozone concentration during the test period is 21.7 ppb while the mean outdoor ozone concentration is 48.4 ppb. The mean indoor PM2.5 concentration during test period is 34.0 μg/m^3^ while the mean outdoor PM2.5 concentration is 71.0 μg/m^3^. By contrast, in the window closed status, the mean indoor ozone concentration during the test period is 12.9 ppb while the mean outdoor ozone concentration is 37.8 ppb. The mean indoor PM2.5 concentration during test period is 32.8 μg/m^3^ while the mean outdoor PM2.5 concentration is 71.3 μg/m^3^. The mean values of indoor concentrations in window closed status are generally lower than the mean indoor concentrations in window open status, particularly for ozone.

Linear regressions were conducted to analyze the linear correlation of measured indoor and outdoor concentrations in different window opening statuses in Figure 8. Comparing Figure 8a,b, the slope of indoor PM2.5 with outdoor PM2.5 (0.65) in window open status is larger than the slope of indoor PM2.5 with outdoor PM2.5 (0.55) in window close status. Comparing Figure 8d,e, the slope of indoor ozone (0.38) with outdoor ozone (0.20) in window open status is also larger than the slope of indoor PM2.5 with outdoor PM2.5 (0.85) in window close status. It indicated that whether the pollutant is PM2.5 or ozone, the change rate of indoor pollutant with the outdoor pollutant in a window open status is larger than with a window close status. Comparing Figure 8a,d, the slope of indoor PM2.5 (0.65) is larger than ozone (0.38) in window open status. Comparing Figure 8b,e, the slope of indoor PM2.5 (0.55) is larger than ozone (0.20). It indicated that regardless of the window opening status, the change rate of indoor PM2.5 with outdoor PM2.5 is greater than that of indoor ozone with outdoor ozone. The coefficient of determination (R2) of each case shows that indoor PM2.5 and ozone concentrations have a significant linear correlation with outdoor concentrations, particularly for PM2.5. Compared to the correlation between indoor PM2.5 concentrations and outdoor PM2.5 concentrations, indoor ozone concentrations have relatively lower degrees of correlation, particularly in the case of having the window closed.

The pollutant I/O ratio can directly represent the relationship between indoor and outdoor pollutant concentrations. Table 2 reveals that the measured I/O ratios of PM2.5 and ozone during the 37 days are in the range of 0.42–0.79 and 0.21–1.00, respectively. Among them, the mean values of I/O ratio of PM2.5 and ozone are lowest in the case of window closed, while the mean values are highest in the case of window open. The I/O ratio is closely associated with the value of ACH in the cases of different window opening status. The value of ACH measured in the case of window open is 1.32, and the value is 0.66 in the case of having the window closed. Moreover, the window open air change rate and window closed air change rate in dormitories in other articles are 1.0–2.1 and 0.2–1.2 respectively [39,86]. The air change rate in this article, the open air change rate and window closed air change rate are 1.23 and 0.49 1/h respectively in the range of the experienced range value. The I/O ratios of PM2.5 are generally higher than the I/O ratios of ozone, even in the case of having the window closed. The high I/O ratio of PM2.5 in the case of having the window closed (0.49) indicates a stronger penetration ability of PM2.5 through the building envelope structures (cracks and wall cavities). The I/O ratios measured in this research are generally consistent with the data measured in previous studies [31,34].

## 4. Conclusions

This study aims to investigate the ventilation and air quality in student dormitories in China. A questionnaire survey and field measurements were conducted in a student dormitory during a summer in Nanjing. The questionnaire survey was performed in 8 dormitory buildings in Nanjing University in the summer of 2013 and 353 valid questionnaires were obtained. According to the survey results, most students thought the indoor and outdoor air quality was neutral in the dormitories. The correlation between indoor and outdoor perceived air quality is statistically significant, although the indoor perceived air quality is slightly related to the outdoor perceived air quality. The survey concerning indoor pollutant source and sink reveals that there are few indoor PM2.5 and ozone sources in most student dormitories. The number of students in the dormitories and their behavior activities are probably the determinant factor influencing the indoor PM2.5 and ozone emission and removal in the dormitories. Besides, natural ventilation is the most common ventilation source in most surveyed dormitories. Most students open the windows sometimes or almost all day. However, there is no statistically significant correlation between the frequency of opening window and the indoor and outdoor perceived air quality during the surveyed period. Most students tend to close the windows in their dormitories during the sleeping period in summer (from 22:00 to 9:00). Besides, more students are inclined to open the windows in the dormitories during daytime and early evening (from 9:00 to 22:00). Particularly, the period between 9:00 and 11:00 is the period with the highest frequency with window open in student dormitories in a day. It is generally consistent with the recorded window opening status during the test period in this study.

Indoor and outdoor PM2.5 and ozone concentrations were monitored in a student dormitory for a period of 37 days in Nanjing University in the summer of 2015. In total, 888 sets of available hour average of PM2.5 and ozone concentration were monitored, including 350 sets in a window open status and 538 sets in a window closed status. Whether the window is open or not, indoor levels of PM2.5 and ozone are generally lower than the outdoor levels and the indoor concentration exceeds the limitation far less often than the outdoor concentration, especially for PM2.5. The survey results reveal that most students often open window between 9:00 to 1:00. The outdoor ozone around 40–80 ppb between 10:00 and 12:00, the highest outdoor ozone concentration can reach 140 ppb. The daily concentration of PM2.5 is stable and its average and highest concentration is 75 μg/m^3^ and 150 μg/m^3^ between 9:00 to 12:0, respectively. Therefore, the ozone concentration is the main problem to be considered. Considering window opening behaviors, the mean values of indoor concentrations in a window closed status are generally lower than the mean indoor concentrations in a window open status, particularly for ozone during 9:00 to 12:00. It indicates that student should stay indoors and minimize window opening in the summer from 10:00 to 12:00. The outdoor and indoor ozone concentrations is at a low level from 7 p.m. to 8 a.m. and this is the most advantageous time to open the window while reducing indoor exposure to severe outdoor ozone pollution in the summer.

In the window open status, the mean indoor ozone concentration during the test period is 21.7 ppb while the mean outdoor ozone concentration is 48.4 ppb. The mean indoor PM2.5 concentration during test period is 34.0 μg/m^3^ while the mean outdoor PM2.5 concentration is 71.0 μg/m^3^. By contrast, in the window closed status, the mean indoor ozone concentration during the test period is 12.9 ppb while the mean outdoor ozone concentration is 37.8 ppb. The mean indoor PM2.5 concentration during test period is 32.8 μg/m^3^ while the mean outdoor PM2.5 concentration is 71.3 μg/m^3^. The mean values of indoor concentrations in the window closed status are generally lower than the mean indoor concentrations in the window open status, particularly for ozone. According to the linear regression analysis, indoor PM2.5 and ozone concentrations have a significant linear correlation with outdoor concentrations, particularly for PM2.5. The measured I/O ratios of PM2.5 and ozone during the 37 days are in the range of 0.42–0.79 and 0.21–1.00, respectively. The I/O ratios for PM2.5 and ozone are 0.49 (±0.05) and 0.26 (±0.05) in the case of window closed (0.66 ACH), and the I/O ratios for PM2.5 and ozone are 0.65 (±0.08) and 0.50 (±0.15) in the case of window open (1.32 ACH). The outdoor and indoor ozone concentrations show prominently diurnal periodic variations, while the PM2.5 concentrations do not show prominently periodic variations during the studied period.

## Figures and Tables

**Table 1:** Occupant perceptions and behavior patterns in the dormitory.

No.	Question	Options
1	How do you feel about the general air quality inside your dormitory?	a. Very good; b. Good; c. Neutral; d. Poor; e. Very poor;
2	How do you feel about the general air quality outside your dormitory?	a. Very good; b. Good; c. Neutral; d. Poor; e. Very poor;
3	Does any of your roommates commonly smoke in your dormitory?	a. Yes; b. No; c. Uncertain;
4	Do you have an air cleaner ^1^ in your dormitory?	a. Yes; b. No; c. Uncertain;
5	Which kind of ventilation do you usually use in your dormitory?	a. Natural; b. Mechanical ^2^; c. Uncertain;
6	How frequent do you usually open the window in your dormitory?	a. Almost all day; b. Sometimes; c. Almost never;
7	When do you usually open the window in your dormitory?	a. 5:00–9:00; b. 9:00–11:00; c. 11:00–14:00; d. 14:00–16:00; e. 16:00–22:00; f. 22:00–5:00; g. Uncertain;

^1^ The type of air cleaner was not specified. ^2^ Mechanical ventilation indicates the air exhaust.

**Table 2:** I/O ratios of PM2.5 and ozone in the case of window closed and open.

Pollutant	PM2.5			Ozone		
Situation	Window Open	Window Closed	Overall	Window Open	Window Closed	Overall
Mean (SD)	0.65 (0.08)	0.49 (0.05)	0.60 (0.10)	0.50 (0.15)	0.26 (0.05)	0.43 (0.17)
Mid-value	0.66	0.49	0.62	0.47	0.25	0.42
Range	0.45–0.79	0.42–0.69	0.42–0.79	0.23–1.00	0.21–0.39	0.21–1.00
